# Expression Quantitative Trait Loci and The Phenogen Database

**Published:** 2008

**Authors:** Laura Saba, Paula L. Hoffman, Cheryl Hornbaker, Sanjiv V. Bhave, Boris Tabakoff

**Keywords:** Genetic theory of alcohol and other drug use, microarray technologies, microarray analysis, phenotype, candidate gene, qualitative trait locus (QTL), expression qualitative trait locus (eQTL), gene expression, gene transcription, genetics, genomics, transcriptomics, high-throughput analysis, messenger RNA (mRNA), brain, laboratory mice, laboratory rats, PhenoGen Database

Researchers from a wide variety of backgrounds and with a broad range of goals have utilized high-throughput screening technologies (i.e., microarray technologies) to identify candidate genes that may be associated with an observable characteristic or behavior (i.e., phenotype) of interest. However, the initial microarray analyses typically also yield many genes that are not related to the phenotype of interest. Therefore, additional analyses are necessary to select the most likely candidates and eventually identify one or more genes that actually underlie that phenotype. After briefly explaining how microarray data are generated, this article describes one approach to narrowing down the resulting candidate genes and a database that can help in this analysis.

## Generation of Data Through High-Throughput Analyses

When the genetic information encoded in DNA is used by the cell to produce the corresponding proteins, the first step in this process involves the “transcription” of a gene into an intermediary molecule called messenger RNA (mRNA). Although all cells in an organism contain the same genetic information, not all genes are actively transcribed in all cells or at all times, and certain regions in DNA (i.e., regulatory elements) help coordinate which gene is transcribed at what time (i.e., regulate gene expression). The entirety of all mRNAs found in a cell, tissue, or organism at a given time is called the transcriptome. Transcriptomics is a research area concerned with the large-scale analysis of the mRNA expression in a given cell, organ, or organism. Transcriptomics technology has flourished in recent years. This is related at least in part to the development of microarray technologies, which allow for high-throughput analyses of gene expression. Microarrays consist of a series of thousands of microscopic spots, each containing a minute amount of a specific DNA sequence (e.g., a short section of a gene or other DNA element), that are used as probes to analyze an RNA sample.

Like experimental technologies, techniques for analyzing the data generated from the microarray technologies continue to evolve. Initially, researchers obtained data for several thousand genes but on a relatively small number of subjects. After applying the appropriate statistics and multiple comparison adjustment, the investigators would compile from these a list of potential candidates that could contribute to the phenotype under investigation. In many cases, this list would contain several hundred genes. For a researcher who is looking to take a few candidate genes to the next step of testing, such a long list was problematic. With only limited resources and time available, the researcher was forced to pick some “favorites” from the list for further testing. More recently, however, techniques have been developed to systematically narrow these lists. These approaches incorporate biological reasoning to avoid a subjective choice of candidate genes. The following section describes one of these strategies.

## Behavioral and Expression Quantitative Trait Loci for Selecting Candidate Genes

One method to identify the most promising candidate genes among the plethora of genes detected during initial microarray screening involves filtering the list of candidate genes by looking for overlap between those areas in the genome that control the phenotype of interest (i.e., behavioral quantitative trait loci [bQTL] ^1^) and regulatory elements in the genome that control the mRNA transcription level of the candidate genes (i.e., expression QTLs [eQTL]). It is important to note, however, that typical QTL analyses are not precise enough to allow investigators to directly identify the actual causal genes or regulatory elements (i.e., loci). Instead, this approach identifies regions of the genome that are likely to contain those causal loci.

Genetic genomics—the study of how the genome controls transcription (Jansen and Nap 2001)—utilizes the QTL methodology to identify regions of the genome that are associated with gene transcription levels (eQTL). These eQTL give insight into mechanisms underlying the control of gene expression. In general, regulatory mechanisms fall into two categories:

- *Cis*-(or local) regulatory mechanisms: A gene is *cis*-regulated if its eQTL region includes the physical location of the gene. Such an eQTL may reflect a variation (i.e., polymorphism) located within the DNA region directly in front of the transcription start point (i.e., the promoter region) or other regulatory regions of the gene.
- *Trans-*(or distal) regulatory mechanisms: A gene is *trans*-regulated if its transcription level is controlled by an eQTL that is located away from the gene’s physical location. In their study of eQTL, Chesler and colleagues (2005) suggested that *trans*-regulating eQTL may represent polymorphisms within transcription factors or other proteins associated with gene transcription through more complex molecular networks. Other researchers have identified *trans*-acting eQTL that coregulate several transcripts (e.g., Bystrykh et al. 2005).

The approach of searching for overlapping bQTL and eQTL is mainly driven by the hypothesis that if a complex behavioral or physiologic phenotype is associated with the expression level of a specific gene as well as with a particular genetic locus (bQTL) then the eQTL for the gene also should be associated with that locus. For example, if functional tolerance to alcohol in mice is associated with a region on Chromosome 3, then one would try to identify a gene whose mRNA transcription level not only is correlated with functional tolerance but also is associated with the same region on Chromosome 3 (i.e., whose eQTL is within that region). By using such a relationship, investigators can filter candidate gene lists and focus on those genes that most likely are associated with a causal relationship. A key drawback to this approach is that it focuses on eliminating false-positive results—that is, differences in gene expression that in fact are not causally related to the behavioral phenotype under investigation. Therefore, such gene expression–based approaches ignore other mechanisms through which a gene could contribute to the phenotype studied (e.g., changes in gene sequence that alter the function of the resulting protein rather than expression levels or mechanisms whereby the initial mRNA transcribed from the gene is processed in different ways to yield different proteins). To detect such effects will require different sets of data.

## Analyzing QTL Data: The PhenoGen Database

To be able to reliably detect eQTL, researchers require large datasets. Given the expense of generating such expression data, it is essential that available data be openly shared across the scientific community (Geschwind 2001; Insel et al. 2003). The PhenoGen database (http://phenogen.uchsc.edu; [Bhave et al. 2007]), which is sponsored by the National Institute on Alcohol Abuse and Alcoholism (NIAAA), the University of Colorado-Denver School of Medicine, Department of Pharmacology, and the Integrative Neuroscience Initiative on Alcoholism (INIA), shares brain microarray data from large panels of inbred, recombinant inbred, selected lines, and genetically modified mice and rats as well as eQTL databases for mice and rats. In addition, this project provides a central Web site with tools to facilitate the analysis and interpretation of microarray data. In this database, several brain gene expression datasets are publicly available. These include (1) a panel of 20 inbred mouse strains, (2) a panel of 30 recombinant inbred mouse strains (BXD), and (3) a panel of 26 recombinant inbred rat strains (HXB/BXH). eQTL for the BXD panel and the HXB/BXH panel also have been calculated, and this information is available to the public to determine overlap between eQTL and bQTL for a given set of genes.

The PhenoGen Web site contains an expansive set of tools for analysis with the functions necessary to go from raw expression data to a well-understood gene list without leaving the site. It also handles different entry and exit points within this workflow. The table contains a list of tools available to users entering the site with different types of data or at different points in the pathway from raw data to candidate gene(s).

## Conclusion

Lists of candidate genes from traditional microarray studies can be large and cumbersome when looking to proceed to the next level of candidate gene studies. Applying such biologically relevant filters such as eQTL/bQTL overlap allows researchers to narrow down candidate gene lists to a more manageable size without introducing subjective bias. The PhenoGen Web site has both the data and tools available for such analysis plus many more types of transcriptome studies.

## Figures and Tables

**Table:** **What Can I Do on the PhenoGen Web site (http://phenogen.uchsc.edu)?**(This Table lists just a sample of the options available to users, depending on their interest in a particular type of data analysis. For more detail, see Bhave et al. 2007.)

**If you have your own microarray data, you can:** Perform quality control and normalization Create a list of differentially expressed (associated) genes using a variety of statistical methods Cluster expression data by samples and/or genes Submit your experiment to Array Express Share your array data with other investigators View the expression level of all probes associated with a particular gene in your array data
**If we have microarray data that you are interested in, you can:** Create an in-silico experiment with array data of your choice Create lists of differentially expressed genes from your in-silico experiment View the expression level of a particular gene in our array data Cluster expression data by samples and/or genes
**If you have phenotype data, you can:** Correlate your phenotype data with expression data from one of our three inbred rodent panels to create a candidate gene list Correlate your phenotype data with your expression data to create a candidate gene list
**If you have a list of candidate genes, you can:** Get a wide variety of annotation information Search PubMed for literature about the genes and specifically co-citations Filter a list of candidate genes using bQTL/eQTL overlap (PhenoGen provides eQTLs, you provide bQTLs) Identify homolog genes in other species Find common transcription binding sites and motifs or simply retrieve upstream sequences Compare multiple gene lists Cluster expression data by samples and/or genes Create a heat map to visualize clustering by both samples and genes Find the expression level of your genes in any set of array data on the Web site Share your list with other investigators

## Footnotes

1 Quantitative traits are characteristics such as height or sensitivity to alcohol that differ in the extent to which an individual possesses that characteristic. These traits generally are determined by more than one gene as well as by environmental factors. The DNA regions that are associated with a quantitative trait are called QTLs.
